# Biohydrogen Production: Strategies to Improve Process Efficiency through Microbial Routes

**DOI:** 10.3390/ijms16048266

**Published:** 2015-04-14

**Authors:** Kuppam Chandrasekhar, Yong-Jik Lee, Dong-Woo Lee

**Affiliations:** School of Applied Biosciences, Kyungpook National University, Daegu 702-701, Korea; E-Mails: chanduibt@gmail.com (K.C.); yjlee75@knu.ac.kr (Y.-J.L.)

**Keywords:** biohydrogen, dark fermentation, photofermentation, bioenergy, renewable resources

## Abstract

The current fossil fuel-based generation of energy has led to large-scale industrial development. However, the reliance on fossil fuels leads to the significant depletion of natural resources of buried combustible geologic deposits and to negative effects on the global climate with emissions of greenhouse gases. Accordingly, enormous efforts are directed to transition from fossil fuels to nonpolluting and renewable energy sources. One potential alternative is biohydrogen (H_2_), a clean energy carrier with high-energy yields; upon the combustion of H_2_, H_2_O is the only major by-product. In recent decades, the attractive and renewable characteristics of H_2_ led us to develop a variety of biological routes for the production of H_2_. Based on the mode of H_2_ generation, the biological routes for H_2_ production are categorized into four groups: photobiological fermentation, anaerobic fermentation, enzymatic and microbial electrolysis, and a combination of these processes. Thus, this review primarily focuses on the evaluation of the biological routes for the production of H_2_. In particular, we assess the efficiency and feasibility of these bioprocesses with respect to the factors that affect operations, and we delineate the limitations. Additionally, alternative options such as bioaugmentation, multiple process integration, and microbial electrolysis to improve process efficiency are discussed to address industrial-level applications.

## 1. Introduction

The growing gap between the energy demand of the world and an insufficient energy supply has caused a steep increase in fossil fuel use. As a result, we encounter the severe constraints imposed by an alarming increase in pollution levels around the world along with the depletion of fossil fuels. Additionally, the continuous increase in the levels of greenhouse gases (GHGs) released from the combustion of fossil fuels aggravates the problems of global warming. Currently, the CO_2_ concentration exceeds 350 parts per million (ppm) by volume, and the increase in concentration potentially increases the greenhouse effect, which results in increasing global temperatures [1,2]. In recent decades, the organic carbon released by human activities is equivalent to that which was accumulated over millions of years. The limited availability of global fossil fuel reserves and concerns about global climate change from GHG emissions prompted notable interest in the investigation and development of eco-friendly, renewable energy alternatives to fulfill the growing energy demands [3]. Therefore, in the current global energy scenario, the diversification of energy and fuel options is an essential requirement [4]. To diversify, bio-based energy is a sustainable and promising alternative to fossil fuel-based energy; this alternative energy can defend against a crisis in the energy supply and can protect the world from the approaching environmental calamity. Recently, global attention focused on hydrogen (H_2_) gas as one of the most promising, eco-friendly, and renewable energy sources. H_2_ is a potentially versatile energy currency that could alter the use of liquid fossil fuels because the fuel has a high-energy yield per unit mass of 122 kJ/g, which is 2.75-fold higher than that of hydrocarbon fuels [5,6]. Additionally, the combustion of H_2_ with O_2_ produces water (H_2_O) as the only by-product, an obviously favorable outcome for a reduction in GHG emissions. In particular, H_2_ is the pre-eminent choice for an energy carrier because it is more similar to electricity than fossil fuels in the framework of energy systems [7].

**Figure 1:**
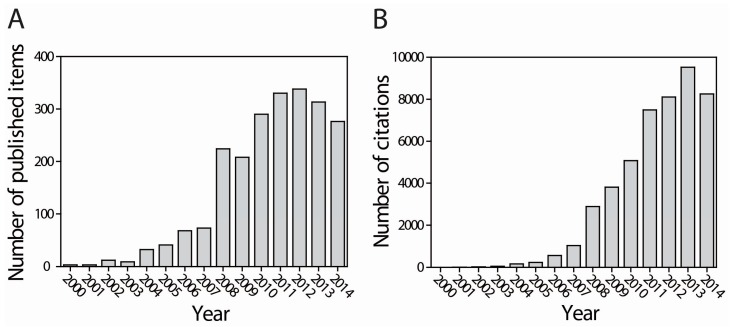
Scientometric analysis of the research on H_2_ production. Published items (**A**) and citations (**B**) in each year.

Currently, molecular H_2_ is primarily produced from the use of fossil fuels through steam reforming of natural gas or methane (CH_4_). The worldwide production of H_2_ currently exceeds 1 billion m^3^/day of which 48% is produced from natural gas, 30% from oil, 18% from coal, and the remaining 4% is produced from H_2_O-splitting electrolysis [2,8]. In combination with steam reforming, the production of pure H_2_ is also achieved with an H_2_O-gas shift reaction, which is one of the important industrial reactions used specifically for ammonia production. The other thermochemical methods available for the production of H_2_ include thermal decomposition, autothermal reforming, catalytic oxidation, pyrolysis, and steam gasification [2,9,10]. However, the production of H_2_ based on fossil fuel resources increases the emissions of GHGs. Alternatively, the production of H_2_ from biomass through biological pathways is an emerging technology because it is sustainable and eco-friendly. Indeed, a scientometric analysis that used the SCI-expanded (since 1994), science technology (CPCI-S, since 1994), and social science (CPCI-SS, since 1994) databases in the ISI Web of Knowledge (Thomson Reuters) found that 2204 research articles were published on H_2_, with a significant number of citations (46,723) and average citations per item (21.02), and a high H-index (92). As shown in Figure 1, the literature linked to H_2_ research increased sharply after 2003 and reached the maximum number of records of 230 in 2008 (total citations: 2883), which was followed by a sudden increase in 2012 (records, 335; citations, 8107). The average citations per year also increased year by year, which clearly indicated that the rapid and promising research continues to make the process of H_2_ production technologically viable.

Different organisms yield H_2_ under specific conditions, including microalgae that use light energy to split water molecules to produce H_2_, and cyanobacteria that typically consume carbohydrates to store energy from photosynthesis to produce H_2_ from water molecules [11,12]. Although there are striking advantages, the low production rates, low substrate conversion efficiencies, and production and accumulation of acid-rich intermediate metabolites from the acidogenic process are practical hindrances that must be overcome for the successful biological production of H_2_. To overcome these limitations, many research projects on the biological production of H_2_ are in progress, and numerous novel approaches are being studied to address some of the existing problems and to overcome these problems by increasing the efficiency of the process. To reach these goals, a number of advanced well-described technologies for high yields of molar H_2_ use metabolic engineering to provide metabolic energy to exceed thermodynamic limitations, to reroute metabolic pathways to increase substrate utilization by the expression of heterologous proteins, and to improve the electron flux for H^+^ reduction, among others [11,12]. In this review, we evaluate the biological pathways for the production of H_2_ with respect to the factors that affect operations and potentially limit the production of H_2_, and assess the efficiency and practical applicability of these technologies. Additionally, alternative options such as bioaugmentation, multiple process integration, and microbial electrolysis to improve process efficiency are discussed.

## 2. Biohydrogen

### 2.1. Diversity of Microorganisms as H_2_ Producing Biocatalysts

**Figure 2:**
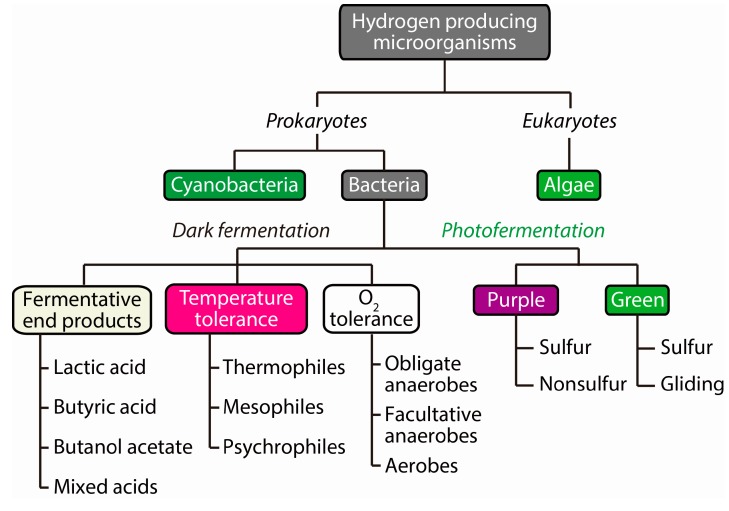
Schematic representation of the diversity of H_2_ producing biocatalysts.

In nature, a variety of organisms including the archaea, anaerobic and facultative aerobic bacteria, cyanobacteria, and lower eukaryotes (*i.e.*, green algae and protists) produce H_2_ [12,13], which may function singly or as a consortium of similar types or mixed cultures (Figure 2). The major H_2_ producing biocatalysts are typical heterotrophs in the fermentation process. Some dark fermentative bacteria do not require solar energy as an energy source and tolerate O_2_ deficient conditions; these microorganisms are obligate anaerobes, which are further classified based on their sensitivity to O_2_ and their growth temperature (Figure 2).

Practically, the culture and maintenance of facultative anaerobes are more feasible than for obligate anaerobes. Based on their growth temperatures, these microorganisms are further classified into mesophiles and thermophiles. Although the thermophiles are cultivated at elevated temperatures with highly intensive energy requirements [14], their H_2_ production can be closer to the theoretical yield than with mesophiles by overwhelming the thermodynamic barrier. Some photofermentative bacteria require light energy to produce H_2_ in anoxygenic conditions. In the absence of O_2_, these photoautotrophs, which include cyanobacteria and green algae, produce H_2_ through biophotolysis using their specific metabolic routes advantageously under defined conditions.

**Figure 3:**
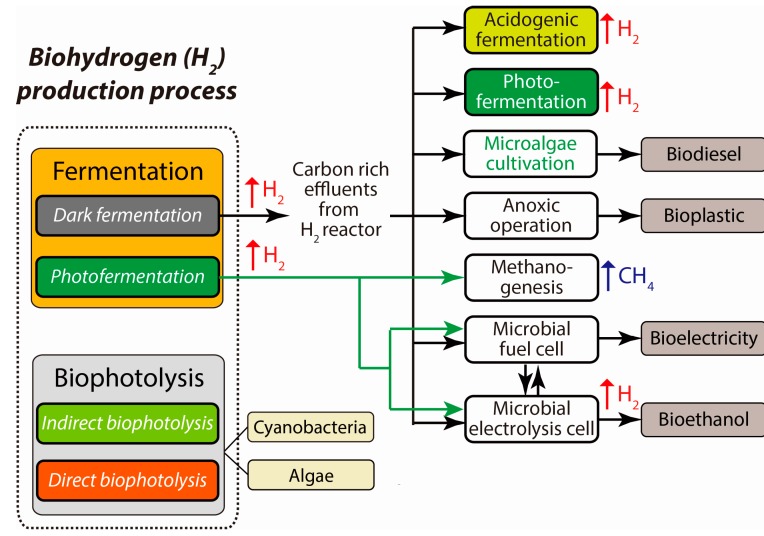
Schematic representation of the primary biological routes integrated with various secondary processes for effective H_2_ production.

Briefly, based on the systems that evolve H_2_, a large number of different natural biological processes are categorized into four primary groups: (1) water-splitting photosynthesis; (2) photofermentation; (3) dark fermentation; and (4) microbial electrolysis processing (Figure 2 and Figure 3). For energy efficiency and practicality, each process has advantages and disadvantages when compared with the other methods. Accordingly, the selection of an appropriate biocatalyst and/or inoculum is an important choice, one that is directly correlated with H_2_ production. Naturally, H_2_ is produced either by a single microbial species or by a mixed consortium of species of which some are involved in the production of H_2_, and the rest consume the H_2_ for their energy requirements. Initial research on H_2_ was typically confined to the use of pure cultures as a biocatalyst with a defined substrate as the carbon source. However, when wastewater was used as the substrate, a mixed microbial population was favorable and practical for application to the scaled-up production of H_2_ [15]. Additionally, mixed cultures are typically preferred because of operational ease, stability, diversity of biochemical functions, and the possibility to use a wide range of substrates [16,17,18]. Therefore, for the practical microbial production of H_2_ in the near future, the proper choice of a H_2_-evolving biosystem together with a deep understanding of the biochemical and biophysical characteristics of the system is a key requirement.

### 2.2. Water-Splitting Photosynthesis (Biophotolysis)

**Figure 4:**
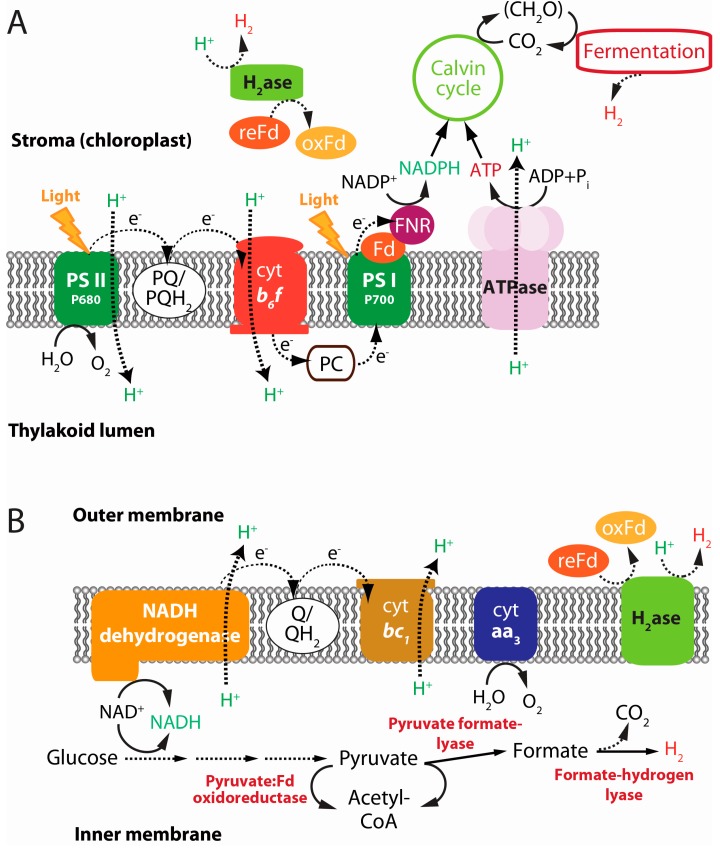
Schematic illustration of H_2_ evolution through (**A**) direct/indirect biophotolysis and (**B**) dark fermentation: (**A**) PS II, photosystem II; PQ, plastoquinone; PQH_2_, plastoquinol; cyt *b*_6_*f*, cytochrome *b*_6_*f* complex; PC, plastocyanin; PS I, photosystem I; Fd, ferredoxin; and FNR, ferredoxin-NADP^+^ reductase. Approximately half of the evolved H_2_ is from water splitting, and the rest of the H_2_ is produced with e^−^ made from the fixed carbon by the activity of the PS I; (**B**) Q, quinone; QH_2_, quinol; cyt *bc*_1_, cytochrome *bc*_1_ complex; and cyt aa_3_, the cytochrome aa_3_ oxidase.

Currently, the most desirable and attractive H_2_ production process is water-splitting photosynthesis, or biophotolysis. The oxygenic photosynthetic microorganisms such as green microalgae (e.g., *Scenedesmus obliquus*, *Chlamydomonas reinhardtii*, *Chlorella*, and *Scenedesmus*, among others) and cyanobacteria (e.g., *Anabaena variabilis*, *Nostoc punctiforme*, and *Synechocystis* sp., among others) use this process that requires only water and sunlight. A (FeFe)-hydrogenase in green algae drives the evolution of H_2_, whereas nitrogenase is responsible for this process in heterocystous cyanobacteria. The biophotolysis is further divided into direct and indirect processes (Figure 3). As shown in Figure 4A, in direct biophotolysis, the electrons derived from the light energy-mediated water splitting are transferred through photosystem II (PS II) and photosystem I (PS I) to ferredoxin (Fd) as an electron carrier, and subsequently, the reduced Fd reduces a hydrogenase enzyme that is responsible for H_2_ production [19]: 2H^+^ + 2Fd(re) ↔ H_2_ + 2Fd(ox). In the case of indirect biophotolysis, photosynthesis converts light energy to chemical energy in the form of a carbohydrate, which is reused to produce H_2_, and at present, these H_2_ producing systems are being intensively investigated using green algae and heterocystous cyanobacteria [12,20]. Because the production of H_2_ by cyanobacteria occurs in the heterocyst, and the oxygenic photosynthesis is microscopic indirect biophotolysis, which is concomitant with CO_2_ fixation in the vegetative cell, the highly O_2_-sensitive nitrogenase is protected, resulting in the production of H_2_: N_2_ + 8e^−^ + 8H^+^ + 16ATP → 2NH_3_ + H_2_ + 16ADP + 16Pi. However, H_2_ production by (FeFe)-hydrogenase and oxygenic photosynthesis cannot occur simultaneously in green algae. Thus, to obtain sustainable H_2_ production, elemental sulfur (S°) deficiency, which causes a severe (≈90%) reduction in photosynthesis, occurred with cells grown on acetate, resulting in a drastic decrease in the oxygen production rate coupled with the improved respiration caused by the existence of residual acetate. In this condition, the cells grow in anaerobic conditions to produce H_2_ by using some of the electrons from the residual water-splitting mechanism (direct biophotolysis) and the reserved carbon (indirect biophotolysis) [21,22].

Recent improvements also include the first direct demonstration of an indirect biophotolysis process that used the nitrogenase enzyme in which the nonheterocystous cyanobacterium *Plectonema boryanum* was recycled multiple times through an aerobic, nitrogen-limited stage, which led to glycogen accumulation, and a second anaerobic, H_2_-producing stage [23]. Additionally, sustained H_2_ production by a single-celled, nonheterocystous cyanobacterium *Cyanothece* occurred with growth in medium supplemented with glycerol for respiratory protection [24] or by replacement of the photosynthetically evolved O_2_ with Argon (Ar) gas [2,25]. Nevertheless, before practical applications, biophotolysis-mediated H_2_ production systems require considerable efforts in protein engineering research to develop O_2_-tolerant hydrogenases in green algae or to replace hydrogenase with nitrogenase in cyanobacteria [11]. Recently, other potential strategies to improve H_2_ production were proposed and investigated, including a decrease in the antenna size [26], downregulation or mutation of the PS II proteins [2,27], changes in operational conditions [28], and heterologous expression of hydrogenase and Fd [2,29].

### 2.3. Anoxygenic Photofermentation

Photofermentation also involves the conversion of light energy to biomass with the production of H_2_ and carbon dioxide (CO_2_); often, the relation is nearly stoichiometric. For the process of photofermentation, purple nonsulfur (PNS) photosynthetic bacteria, including *Rhodobacter* species, are used to convert organic acids such as acetate, lactate, and butyrate to H_2_ and CO_2_ in anaerobic and anoxic conditions. Moreover, these bacteria capture solar energy to transform organic acids into H_2_ using nitrogenases in the absence of ammonium (NH_4_) ions [2,30,31]. In particular, O_2_-sensitive nitrogenase is not a problem for this process because the purple bacteria used in the process have nonoxygenic photosynthesis [30]. However, these nitrogenases also possess several defects that affect the production of H_2_, including low catalytic activity, suppression of their expression by NH_4_, and lower photochemical efficacy [32,33]. In theory, the photofermentation process can completely convert organic compounds into H_2_, even reaching a comparatively high H_2_ partial pressure; simultaneously, H_2_ production is driven by nitrogenase, and ATP is formed with the capture of solar energy through a photosynthetic mechanism in these bacteria (e.g., *Allochromatium vinosum*, *Thiocapsa roseopersicina*, *R. sphaeroides*, *Chlorobium vibrioforme*, *Desulfuromonas acetoxidans*, and *Chloroflexus aurantiacus*). Recently, a comparatively high-yield conversion efficacy of sugars to H_2_ was achieved [34], and the completely stoichiometric transformation of glycerol to H_2_ was attained [35,36]. These are light-dependent processes in which captured light energy drives the electron stream through the photosynthetic system, leading to a proton gradient. This proton gradient is further used to fulfill both requirements for nitrogenase activity: ATP, produced with ATP synthase, and high-energy electrons, generated through inverse electron flow.

### 2.4. Dark Fermentation

The dark fermentative process also produces H_2_ (Figure 4B). To date, many of the studies on the biological production of H_2_ through the dark fermentation process were performed using facultative (e.g., *Enterobacter aerogenes*, *E. cloacae*, *Escherichia coli*, and *Citrobacter intermedius*, among others) and obligate anaerobic bacteria (e.g., *Clostridium beijerinckii*, *C. paraputrificum*, and *Ruminococcus albus*, among others) [37,38,39]. The process of dark fermentation occurs at a higher rate than the processes of photofermentation and photolysis. However, the low yield of H_2_ on substrates, because of the formation of various by-products, is the primary disadvantage. The fermentation process helps to generate energy-rich reducing compounds (*i.e.*, NAD(P)H and FADH) from metabolic pathways, which are then sequentially reoxidized by respiratory chains with a terminal electron acceptor (TEA), resulting in the formation of energy-rich molecules (ATP). In aerobic respiration, O_2_ is a TEA that helps to generate ATP with concurrent regeneration of the reducing powers. By contrast, anaerobic respiration uses a variety of organic and inorganic compounds (e.g., NO^3−^ and SO_4_^2−^, among others) as TEAs with their concurrent reduction and regeneration of reducing powers. Glycolysis is the key metabolic pathway in which a substrate can be transformed into pyruvate, a central metabolic intermediate. Under anaerobic conditions, the pyruvate enters into the acidogenic pathway coupled with H_2_ production, which results in the formation of volatile fatty acids (VFAs) (*i.e.*, acetic acid, propionic acid, butyric acid, and malic acid, among others) (Equations (1)–(5)).

C_6_H_12_O_6_ + 2H_2_O → 2CH_3_COOH + 2CO_2_ + 4H_2_ (acetic acid pathway) (1)

C_6_H_12_O_6_ + 2H_2_ → 2CH_3_CH_2_COOH + 2H_2_O (propionic acid pathway) (2)

C_6_H_12_O_6_ → CH_3_CH_2_CH_2_COOH + 2CO_2_ + 2H_2_ (butyric acid pathway) (3)

C_6_H_12_O_6_ + 2H_2_ → COOHCH_2_CH_2_OCOOH + CO_2_ (malic acid pathway) (4)

C_6_H_12_O_6_ → CH_3_CH_2_OH + CO_2_ (ethanol pathway) (5)

As stated above, both obligate and facultative bacteria produce H_2_using a wide variety of organic substrates [11]. Facultative anaerobic microorganisms transform pyruvate to acetyl-CoA and formate with the catalysis of pyruvate formate-lyase, and then produce H_2_ with formate hydrogen lyase [40]. However, obligate anaerobic microorganisms convert pyruvate to acetyl-CoA and CO_2_ with pyruvate ferredoxin oxidoreductase. This process of oxidation requires the reduction of Fd [41]. During fermentation, the H^+^ reducing mechanism facilitates the production of H_2_ as a by-product. The interconversion of metabolic intermediates occurs during substrate consumption in anaerobic fermentation, which increases the availability of reducing equivalents in bacterial cells [42,43]. The H^+^ released from NADH/FADH with NADH dehydrogenase is reduced to H_2_ by hydrogenase with the reduced Fd, whereas the membrane-bound protein complexes (*i.e.*, NADH dehydrogenase and cytochrome *bc*_1_) and the mobile electron carriers (quinone (Q) and cytochrome *c*) facilitate the electron transfer through the Q pool. The continuous redox interconversion between Q (QH_2_) and H^+^ conveys electrons to the cytochrome *bc*_1_ complex (cyt *bc*_1_) and to the cytochrome aa_3_ (cyt aa_3_). Consequently, the cyt aa_3_ reduces the Fe-S containing Fd, which donates an electron to the hydrogenase responsible for H_2_ production [2,40,43].

### 2.5. Microbial Electrolysis Cells (Electrofermentation)

Microbial electrolysis cells (MECs), a new technique to produce H_2_ from a wide variety of substrates, have been rapidly developed in the last few years. These are fundamentally adapted microbial fuel cells (MFCs), which have been investigated for decades. Using the MECs as an alternative electrically driven H_2_ production process results in the conversion of a wide range of organic substrates into H_2_ under applied external potential. The MEC technology is also called electrofermentation or biocatalyzed electrolysis cells [44,45]. The MEC technology resembles an MFC in which the primary difference is the necessity of a small input of external voltage. Based on thermodynamics, a potential higher than 0.110 V, in addition to that generated by a microorganism (−0.300 V), will produce H_2_ [44]. The normal redox potential for the reduction of H^+^ to H_2_ is −0.414 V; therefore, the potential requirement is very low when compared with the theoretically required voltage of 1.230 V for the electrolysis of H_2_O [45].

In practice, however, a comparatively higher potential than this value is required because of the over potentials created by physical, chemical, and microbial factors [2,46]. The MECs are capable of more than 90% efficiency in the production of H_2_ [44]. However, the performance of MECs is determined by the type of microorganism, electrode materials, type of the membrane used, applied potential range, composition and concentration of the substrate, and design of the MEC. Initially, the MECs were used in a two-chamber configuration, which was shifted later to a single chamber configuration because of the ease of the process, a significant reduction in internal resistance, a decrease in pH in the anode chamber caused by the production and accumulation of H^+^, and an increase in pH in the cathode chamber caused by the use of H^+^, and the provision of a membrane is avoided [46]. Indeed, the exclusion of a membrane reduced both the pH and the losses of energy and ohmic energy in a single chamber MEC operation [47], which was the primary problem with a two-chamber MEC configuration [45].

## 3. Effects of Operational Factors for H_2_ Production

### 3.1. Temperature

One of the key factors during the fermentation process is the operating temperature because it can alter not only the microbial use of the substrate and the specific growth rate but also the H_2_ production and the metabolic product formation. Several studies reported on dark fermentative H_2_ production at different temperatures: ambient (15–27 °C), mesophilic (30–45 °C), thermophilic (50–60 °C), and extremely thermophilic (>60 °C) [48,49]. The optimal growth temperatures for mesophilic bacteria (e.g., *C. butyricum* and *E. cloacae*) and hyperthermophiles (e.g., *Pyrococcus furiosus* and *Caldicellulosiruptor bescii*) ranged from 30 to 45 °C and from 50 to 80 °C, respectively. However, when mixed cultures were used, the optimal temperature for H_2_ production changed from the optimal growth temperature for each strain [17,48,50]. Most of the H_2_ production studies at the laboratory scale were performed using mesophilic microorganisms because of the ease of operation and the maximum specific growth rates [51]. Tang *et al.* [50] investigated the effects of temperature on H_2_ production with mixed cultures and demonstrated that the maximal yield of H_2_ (319 mL of H_2_/g substrate, measured in the form of COD) was obtained by increasing the temperature from 35 to 45 °C, whereas the yield decreased to 182 mL of H_2_/g substrate as the temperature increased from 45 to 55 °C. Lin *et al.* [52] also performed studies to optimize the temperature for H_2_ production with a mixed microbial population using a chemostat-type H_2_ bioreactor, and the highest H_2_ production was achieved at 45 °C.

Recently, much attention focused on thermophiles as attractive alternatives for the production of H_2_ [53]. Notably, the production of H_2_ benefits from some general advantages that are gained by performing processes at elevated temperatures, such as a lower viscosity, better mixing, less risk of contamination, higher reaction rates, and no need for cooling of the bioreactor. Additionally, the yields of H_2_ using thermophiles could reach the maximum theoretical value of 4 mol H_2_/mol glucose, which is much higher than examples with mesophiles that reached the maximum yield of less than 2 mol H_2_/mol glucose [53,54]. Indeed, operation at high temperatures is thermodynamically favorable for increased H_2_ production because of an increase in the entropy of the system, which makes the system more energetic and avoids contamination of the H_2_-utilizing enzymes and microorganisms [50]. Overall, one of the key factors for H_2_ production is clearly operational temperature, which typically depends on the type of the H_2_-producing microorganism and on the type of substrate used.

### 3.2. pH

The pH of the system (redox microenvironment) is an essential index for the microbial population and is an integral expression of the redox conditions for any anaerobic process [55,56]. Thus, pH has a key role in the regulation of metabolic pathways and in the production of H_2_ because the fermentative metabolites derived from pyruvate determine the yield of H_2_ [57]. Accordingly, changes in external pH values also affect several physiological parameters in cells, such as the internal pH, proton motive force, and membrane potential. As the rate-limiting factor, these results clearly indicate that the pH-dependent activity of the microorganisms directly affects the pH-dependent fermentations associated with H_2_ production [58]. For example, in an acidic microenvironment (low pH), pyruvate is converted to VFAs, concomitant with H_2_ production, whereas a neutral pH leads to CH_4_ production by methanogenic microorganisms [43]. Indeed, H_2_ producing microorganisms function well below a pH of 6, whereas the optimum pH for methanogenic microorganisms is between 6.0 and 7.5. The production of H_2_ was high when the pH was maintained at approximately 6.0 [59], but an extremely acidic microenvironment (pH < 4.5) was detrimental to the ability of the microbial community to produce H_2_ [58,60]. In an alkaline microenvironment, fermentative pathways are prone to solventogenesis [18]. Additionally, the H^+^ that shuttles among metabolic intermediates causes the formation of reduced compounds (e.g., aldehydes, alcohols, and reducing sugars) and altered membrane potentials, which results in decreased cellular growth rates [61,62]. The accumulation of VFAs during the fermentation process leads to a decrease in the system pH, which decreases the system buffering capacity and, ultimately, inhibits H_2_ production [18,60,63]. The pH range of 5.5–6.0 might be beneficial for the production of H_2_ with dark fermentation and for the prevention of methanogenesis and solventogenesis [18].

### 3.3. Nutrients

In the presence of a carbon (C) source, the supplementation with nutrients for bacterial growth is also critical to increase H_2_ production. The study of Lin and Lay demonstrated that at a carbon/nitrogen (C/N) ratio of 47, the productivity and the production rate of H_2_ reached 4.8 mol H_2_/mol sucrose and 270 mmol H_2_/L per day, respectively [63]. In a mixed culture, the maximum H_2_ production potential of 291.4 mL (corresponding to 3.25 mmol), the maximum H_2_ yield of 298.8 mL/g of glucose (3.33 mmol H_2_/g of glucose), and the maximum average H_2_ production rate of 8.5 mL/h were obtained at the ammonia concentration of 0.1 g of N/L [64]. However, excess amounts of nitrogen affected the intracellular pH of the microorganism used for the production of H_2_ and also inhibited the activity of nitrogenase [65,66]. Additionally, high concentrations of N induced ammonification, which was not favorable for H_2_ production [67]. An optimum C/N ratio influenced the microbial growth rate and H_2_ yield in mixed or pure cultures; however, it also adds to the overall production costs [68]. Thus, the research is in progress to find alternative sources of N in the substrate. For example, the wastes of the corn starch manufacturing process (corn-steep liquor) offer a promising alternative supplementation for peptone [69]. The optimal concentrations of phosphate (P) are also essential to increase the yield of H_2_ in which P is an important inorganic nutrient for the fermentative production of H_2_ [63]. Phosphorus, in the form of adenosine triphosphate (ATP), has a major role in energy generation in the bacterial cell and is also involved in the system that regulates the buffering capacity as an alternative to carbonate [42]. Nevertheless, a high concentration of P stimulates excessive production of VFAs, which leads to significant decrements in the yield of H_2_ with the diversion of cellular reductants away from the production of H_2_.

For any fermentative process, supplementation with suitable metal ions is necessary to activate many of the enzymes and coenzymes that are related to microbial metabolism and cellular transport processes and that are also essential for cell growth. Each of the metal ions has a precise function in the cell during metabolism, and a change in their availability may change that function [70]. Hydrogenase, a crucial enzyme in the H_2_ production process, has a bimetallic Fe–Fe center surrounded by Fe-S protein clusters [71]. The iron (Fe) also acts as an active site for the Fd, which transports electrons to the hydrogenase. Consequently, several researchers investigated the consequences of Fe supplementation for H_2_ production. Lee *et al.* [72] investigated the effect of Fe concentration on H_2_ production and observed that high concentrations had a positive effect on the system because the Fe is an essential component of the Fd and hydrogenase [73,74,75]. The optimal concentration of Fe was approximately 25 to 100 mg/L, and higher concentrations than those led to toxicity [73,76]. In another investigation, Lin and Shei [70] examined the influence of different trace elements, specifically Fe, Ni, Mg, Mn, Na, Zn, K, I, Co, Cu, Mo, and Ca, for H_2_ production using *C. pasteurianum* and found that the appropriate concentrations of Mg, Na, Zn, and Fe were essential to achieve the highest yields of H_2_ [70,73,74,77].

### 3.4. Hydraulic Retention Time

The hydraulic retention time (HRT) is also an important factor in the selection of microorganisms because microorganisms are required with growth rates that can withstand the mechanical dilution caused by continuous volumetric circulation. An extended fermentation time is unfavorable for H_2_ production because of the metabolic shift from acidogenesis to methanogenesis. Preferably, a shorter HRT would restrict the growth rate of methanogenic microorganisms [78]. For satisfactory H_2_ yields, the optimum HRTs were between 8 and 14 h for a wide variety of substrates [78,79,80]. By maintaining short HRTs (2–10 h), the methanogenesis was effectively suppressed [81]. However, for the optimum H_2_ yield, the HRT is influenced by several factors, including the type and composition of the substrate, the type of microorganism, the organic loading rate, and the system redox condition, among others. Unlike the dark fermentation process, a short HRT reduces the substrate use efficiency and therefore decreases the process efficiency during photofermentation [82].

### 3.5. Partial Pressure of H_2_

The biological pathways of H_2_ production are highly sensitive to the partial pressure of H_2_ (H_PP_), which is a key rate-limiting factor, particularly during the process of dark fermentation because hydrogenase activity (transfer of an electron from an intracellular electron carrier to H^+^) is likely to decrease because of feedback inhibition [16]. When the level of H_2_ dissolved in the fermentation medium increased, the reduction of oxidized Fd occurred more favorably than the oxidation of reduced Fd, which caused the hydrogenase to be reversibly oxidized and the Fd to be reduced, resulting in a decrease in H_2_ production because of the oxidation of the dissolved H_2_ [18]. The metabolic pathways shift from acidogenesis to solventogenesis to form reduced products, such as lactate, ethanol, acetone, butanol, and alanine, which leads to a decrease in H_2_ yield under higher H_pp_ conditions (greater than 60 Pa) because of thermodynamically unfavorable conditions [83]. The sparging of inert gases (nitrogen and argon, among others) into the reactor headspace in combination with gas stripping to maintain low H_pp_ successfully increased the H_2_ yield by 68% [18,84]. Therefore, operating H_2_ bioreactors at a low H_pp_ leads to high yields of H_2_ [16,18,85].

## 4. Economic Feasibility and Technical Challenges

During the last two decades, several efforts to make the H_2_ production process economically more feasible were attempted [46]. However, some key technical challenges remain, and if these challenges are overcome, the overall H_2_ production efficiency will increase through the biological pathways described below (Table 1).

**Table 1:** Biological pathways for H_2_ production and the technical limitations.

Type of Bioprocess	Technical Challenges
Dark fermentation	low substrate conversion efficiency low H_2_ yield thermodynamic limitations mixture of H_2_ and CO_2_ gases as products, which require separation
Photofermentation	requirement of an external light source the process is limited by day and night cycles, with sunlight as the light source low H_2_ yield caused by extremely low light conversion efficiency
Direct biophotolysis	O_2_ generation caused by the activity of PS II requirement for customized photobioreactors low H_2_ yield caused by extremely low light conversion efficiency
Indirect biophotolysis	lower H_2_ yield caused by hydrogenase(s) requirement of an external light source total light conversion efficiency was very low

These challenges may be overcome with the efficient design of H_2_ producing bioreactors, process modifications, selection of appropriate feedstocks, and with the selection of suitable and efficient microbial strains. In the metabolic pathways that produce H_2_, the intermediate metabolites produced by the biocatalyst compete for the identical reductants as the H_2_, and this redirection of the reductants toward soluble end metabolites reduces the H_2_ yields. Hence, several researchers are attempting to reroute the metabolic pathways to reduce the production of the low-end metabolites. To overwhelm the stoichiometric limitation (4 mol H_2_/mol glucose) of the dark fermentation process, a robust biocatalyst must be found that can be metabolically engineered. Thus, with a successful bioreactor design and the determination of the ideal process parameters, the yield of H_2_ can be increased. However, low molar conversion rates affect the economics of the process, and therefore, research is underway to increase the H_2_ yield above the 4 mol H_2_/mol glucose limitation. Recently, several researchers focused on the development of suitable hybrid processes, such as the two-stage integration of the dark fermentation process, followed by the photofermentative process to produce H_2_ [18]. With this approach, the VFAs that are produced in the dark fermentation (first stage) are used as the substrate in the photofermentation (second stage). This approach might use efficiency to increase the theoretical limit of H_2_ yield to 12 mol H_2_/mol glucose [14]. Similarly, hybrid processes that include subsequent methane production or electrofermentation are also being considered to increase the energy recovery of the process. Although there is an abundance of research in the past and at present, these specific areas must be investigated to further enhance the production of H_2_ via the biological pathways [37]. Additionally, the integration of the H_2_ production process with a conventional wastewater treatment process has several advantages, such as waste remediation with simultaneous generation of clean energy. In the future, carbon-rich organic wastes may be targeted as suitable feedstocks for H_2_ production because of their natural abundance. The use of cheaper raw material substrates would increase the H_2_ yield from the biological processes, which would help significantly to make the process more economically viable and cost effective.

## 5. Strategies to Enhance the Efficiency of the Process

The major deterrents to the conventional biological H_2_ production from any of the processes described above are the low substrate conversion efficiency and the accumulation of VFAs. Because of these deterrents, the overall yield of H_2_ is far too low for the process to be economically feasible and commercially applicable [38,79]. In particular, although the theoretical H_2_ production could reach 12 mol of H_2_/mol glucose, the dark fermentative H_2_ production is metabolically limited to 4 mol H_2_/mol glucose, which is a major technical hurdle for practical applications [86]. Additionally, after dark fermentation, significant amounts of residual organic substances such as VFAs or solvent remain in the effluent. Thus, additional treatments are necessary before disposal into the environment. The reuse of the residual carbon fraction of the fermentative effluents for further energy generation together with proper environmental treatment would be wise considering the environmental and economic factors [87]. Moreover, the design and fabrication of photobioreactors that use the internal light supply efficiently remains a challenge in photofermentation [88,89].

### 5.1. Integration of Approaches

Recently, many integrated approaches were proposed to overcome the limitations of several processes to increase the production of H_2_ in dark fermentation. The use of the residual acid-rich organic substances from the fermentation effluents as carbon-rich substrates for further energy recovery is a viable and novel idea, particularly when in the form of an integrated two-stage energy producing process (Table 2 and Figure 3). Numerous secondary processes, including methanogenesis for methane, acidogenic fermentation for H_2_, photobiological processes for H_2_ [90,91,92], MECs for H_2_ [44], anoxygenic nutrient-limiting processes for bioplastics, cultivation of heterotrophic algae for lipids, and MFCs for bioelectricity generation, were integrated with the primary dark fermentative process of H_2_ production. With these integrated approaches, the primary process uses these further substrates for additional energy production, and therefore, the entire process is more economically viable and practically applicable than without the integration.

**Table 2:** A list of the processes integrated with the production of H_2_ from dark fermentation (DF, dark fermentation; PF, photofermentation; MEC, microbial electrolysis cell; BEH, bio-electrohydrolysis).

Substrate	First Stage		Second Stage		Reference
	Process Type	Yield	Process Type	Yield	
Cornstalks	Hydrogen (DF)	58.0 mL/g	Methane (DF)	200.9 mL/g	[93]
Rice straw	Hydrogen (DF)	20 mL/g	Methane (DF)	260 mL/g	[94]
Water hyacinth	Hydrogen (DF)	38.2 mmol H_2_/L/day	Methane (DF)	29 mmol CH_4_/L/d	[95]
Water hyacinth	Hydrogen (DF)	51.7 mL of H_2_/g of TVS	Methane (DF)	43.4 mL of CH_4_/g of TVS	[96]
*Laminaria japonica*	Hydrogen (DF)	115.2 mL of H_2_/g	Methane (DF)	329.8 mL of CH_4_/g	[97]
Cassava wastewater	Hydrogen (DF)	54.22 mL of H_2_/g	Methane (DF)	164.87 mL of CH_4_/g	[98]
Microalgal biomass	Hydrogen (DF)	135 ± 3.11 mL of H_2_/g/VS	Methane (DF)	414 ± 2.45 mL of CH_4_/g/VS	[99]
Glucose	Hydrogen (DF)	1.20 mmol	Hydrogen (PF)	5.22 mmol	[100]
Cheese whey wastewater	Hydrogen (DF)	2.04 mol	Hydrogen (PF)	2.69 mol	[101]
Vegetable waste	Hydrogen (DF)	12.61 mmol H_2_/day	Electricity (DF)	111.76 mW/m^2^	[87]
Fruit juice industry wastewater	Hydrogen (DF)	1.4 mol H_2_/mol hexose	Electricity (DF)	0.55 W/m^2^	[102]
Corn stover lignocellulose	Hydrogen (DF)	1.67 mol H_2_/mol glucose	Hydrogen (MEC)	1.00 L/L-d	[103]
Cellobiose	Hydrogen (DF)	1.64 mol H_2_/mol glucose	Hydrogen (MEC)	0.96 L/L-d	[104]
Distillery spent wash	Hydrogen (DF)	39.8 L	Bioplastic	40% dry cell weight	[105]
Food waste	Hydrogen (DF)	3.18 L	Bioplastic	36% dry cell weight	[106]
Pea shells	Hydrogen (DF)	5.2 L of H_2_ from 4 L	Bioplastic	1685 mg of PHB/L	[107]
Food waste	Hydrogen (DF)	69.94 mmol	Lipid	26.4% dry cell weight	[108]
Olive oil mill wastewater	Hydrogen (DF)	196.2 mL/g	Biopolymer	8.9% dry cell weight	[109]
Molasses wastewater	Hydrogen (DF)	130.57 mmol	Ethanol	379.3 mg/L	[110]
Food waste	Bioelectricity	85.2 mW/m^2^	Hydrogen (DF)	0.91 L	[39]
Starch hydrolysate	Hydrogen (DF)	5.40 mmol H_2_/g of COD	Hydrogen (PF)	10.72 mmol H_2_/g of COD	[111]
Sucrose	Hydrogen (DF)	0.98 ± 0.32 mol H_2_/mol	Hydrogen (PF)	4.48 ± 0.23 mol H_2_/mol	[112]
Glucose:xylose (9:1); Microalgae biomass	Hydrogen (DF)	250 mL/L/h; 2.78 mol H_2_/mol	Mixotropic microalgae cultivation	205 mL/L/h; 1.12 g of biomass/g of COD	[113]

### 5.2. Photobiological Process

The photosynthetic bacteria readily consumed the residual organic fraction (VFAs) [100,101]. Because dark fermentative metabolic intermediates can be effectively used by some PNS bacteria, the integration of the anoxygenic photofermentation process with the dark fermentation process will have the dual advantages of increased H_2_ production with simultaneous removal of the substrates [114]. Chandra and Venkata Mohan [100] investigated the composition and the survivability of mixed microalgal populations during their growth and the production of photofermentative H_2_ using glucose and acid-rich effluents generated from the process of dark fermentation. Photofermentation with the acid-rich effluents of glucose had a higher efficiency of H_2_ production (5.22 mmol H_2_) than dark fermentation (1.21 mmol H_2_) with glucose as the carbon source. Green algae such as *Chlorella* also use carbon-rich organic acid intermediates from dark fermentation to produce H_2_, particularly when acetate is a viable substrate (Equations (6) and (7)) [115].

Dark fermentation (Stage I):

C_6_H_12_O_6_ + 2H_2_O → 2CH_3_COOH + 2CO_2_ + 4H_2_ (6)

Photoheterotrophy (Stage II):

2CH_3_COOH + 4H_2_O → 8H_2_ + 4CO_2_ (7)

The Equations (6) and (7) define an ideal condition in which all the carbon in the form of substrate is processed in the suitable metabolic pathways and none of the carbon is routed to the formation of biomass or alternative metabolites. However, the photofermentation of acid-rich effluents from the H_2_ production process is more complex than dark fermentation with respect to the efficiency of processing because of poor light penetration, nutritional requirements of the biocatalyst, maintenance of environmental conditions, inhibition of substrates, and contamination obstacles [116,117]. To overcome these limitations, appropriate light arrangements must be either inside or outside of the bioreactor, sufficient nutrients must be supplemented, optimum temperatures and substrate concentrations must be maintained, and the bioreactors must be enclosed systems for ease of sterilization.

### 5.3. Biodegradable Plastics

The VFA-rich effluents generated from dark fermentation are a potential substrate for the production of bioplastics, such as polyhydroxyalkanoates (PHA) and polyhydroxybutyrates (PHB). The PHAs are a biodegradable biopolyester of hydroxyalkanoates that are produced under extra carbon and nutrient-deprived circumstances and that accumulate as cellular reserve storage material [105,106]. The biopolyesters are deposited as water-insoluble, cytoplasmic micro-sized inclusions in bacterial cells when excess carbon is available and when other nutrients are growth limiting. In general, the PHAs are produced using pure microbial cultures with synthetic substrates (e.g., acetate and butyrate, among others), which is not a cost-effective method of production. The VFAs are simple substrates with a low number of carbon atoms, and the synthesis of PHA requires fewer metabolic enzymes than those of the glycolysis and β-oxidation pathways [106]. The production of PHBs from individual fatty acids (e.g., acetate and butyrate, among others) and acid-rich effluents from dark fermentation was reported for an anoxic microenvironment that used a mixed culture as the biocatalyst [118]. Reddy *et al.* [106] investigated the production of bioplastics (PHA) using *B. tequilensis* in aerobic conditions with synthetic acids (SA) and acid-rich effluents of food waste (AFW) as substrates, which were collected from bioreactors producing H_2_ with dark fermentation. The synthesis of PHAs was higher with SA (59% dry cell weight) than with AFW (36% dry cell weight). They also reported on the presence of a copolymer (P(3HB-*co*-3HV)) with varying amounts of hydroxy butyrate (HB, 80%–90%) and hydroxy valerate (HV, 10%–15%) for both substrates. Accordingly, the use of the acid-rich effluents from H_2_ producing reactors as substrates contributed to a significant reduction in the production costs of both the H_2_ and the PHA embedded with the waste valorization. The entire process was more economically viable when the production of bioplastics was coupled with the production of H_2_ and their effluents were used for methanogenesis [107].

### 5.4. Electrically Driven Biohydrogenesis from Acid-Rich Effluents

**Figure 5:**
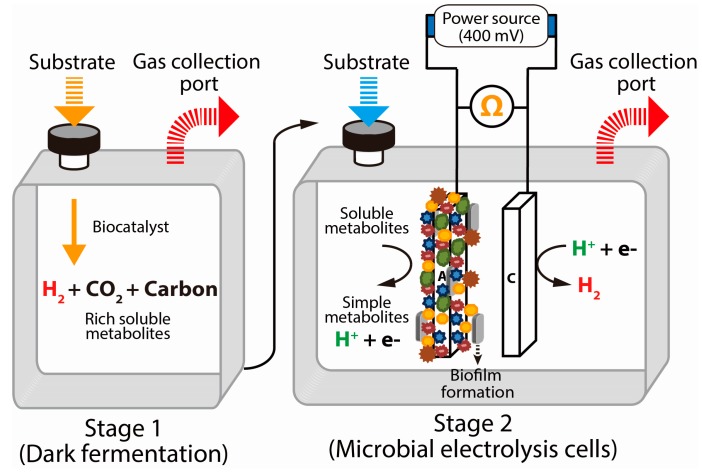
Schematic illustration of microbial electrolysis cells (MECs) integrated with the dark fermentation process for higher H_2_ yield (A: anode; C: cathode; Biofilm: electrochemically active mixed microbial population). Green, orange, brown, and blue symbols represent a mixed microbial population. In stage 1, initially, complex substrates were used for H_2_ production in dark fermentation, and in stage 2, acid-rich effluents were used as substrates in MECs for further H_2_ production.

In recent years, the integration of MECs with other bioprocesses has also received considerable attention [103,119]. As an alternative electrically driven process of H_2_ production, the MECs facilitate the transformation of biodegradable materials into H_2_ with an external voltage applied. Indeed, the MEC process was feasible to generate H_2_ in association with simultaneous wastewater treatment for a wide variety of soluble organic substances [104,120]. A two-stage process was used to convert the acid-rich dark fermentation effluents into substrates for additional H_2_ production (Figure 5) [103,104]. Babu *et al.* [104] investigated the feasibility of integrating the MEC process with the dark fermentation process to use the acid-rich effluents for additional H_2_ recovery. For this integration, the MECs were operated with a small range of varying applied potential (0.2, 0.5, 0.6, 0.8, and 1.0 V) and with acid-rich effluents (concentration of 3000 mg/L) using an anaerobic mixed consortium as a biocatalyst. The maximum hydrogen production rate (HPR) and the cumulative hydrogen production (CHP) were 0.53 mmol/h and 3.6 mmol, respectively, with 49.8% of the VFAs utilized at 0.6 V. With a high substrate conversion efficiency (90%), a two-stage approach, *i.e.*, MECs integrated with dark fermentation, could be a viable option to achieve higher substrate conversion efficiency and H_2_ yield [44].

### 5.5. Bioaugmentation

Many biotic and abiotic factors (e.g., microbial physiology and concentration and composition of substrates) also affect the overall yield of the dark fermentative H_2_ production process with mixed cultures. With the reactor in operation, higher substrate concentrations lead to an accumulation of VFAs and a decrease in system pH (˂4.0), which results in the inhibition of the H_2_ production process. In an effort to improve the process capability with higher substrate conversion efficiencies, the addition of the desired microbial strains to a native microbial community would be a practical option to overcome the inhibition in the process [121]. These bioaugmentation strategies used single or mixed native microflora with the acidogenic consortia [122], fermentative H_2_ producing bacteria [123], and *C. acetobutylicum* [124] to increase H_2_ production efficiency. Moreover, similar studies were performed to recover the start-up of a bioreactor [125], to boost reactor performance [126], and to protect the native microbial community against problems in the process [121], which indicated that the bioaugmentation strategy was effective to increase H_2_ production. In some cases, however, the bioaugmented microbial flora might fail to compete with the native population, most likely because of inappropriate operating conditions, failure of substrate utilization, and type and/or diversity of the native microbial population in the system [127,128,129].

### 5.6. Utilization of Organic Wastes as a Fermentable Substrate

With rapid urbanization and industrialization, waste management is at the forefront as a major human health and environmental concern [42], and improper waste management increases GHG emissions, which contribute to climate change. Moreover, because many effluents and wastes from foods and food processing and industries that use paper, dairy, cellulosic, and glycerol require a high chemical and biological oxygen demand [130], they potentially threaten the aquatic fauna [131]. For practical and economical aspects, the use of carbon-rich wastes/wastewater as fermentable substrates is an attractive and promising approach for H_2_ production, which may solve the dual purpose of waste disposal and clean energy generation [132]. This approach would greatly reduce the H_2_ production processing costs, when compared with chemical and electrolytic processes [133]. The fermentable waste contains biodegradable organic materials to yield a net positive energy, which remains valid for thermophilic fermentative H_2_ production, although this process requires additional energy for the heating of the substrates and the reactor [16]. Moreover, bioreactors may be installed at or proximal to waste generation sites, which further increases the economic viability of the process [62]. Therefore, a variety of wastes could be used as potentially fermentable substrates for H_2_ production.

### 5.7. Pretreatment of Substrates and Biocatalysts

Complex substrates/feedstocks are not the preferred substrates for H_2_ producing biocatalysts and are a challenging bottleneck to the development of biological pathways for H_2_ production. To transform nonutilizable substances into fermentable substrates, many pretreatment processes were assessed to make complex substrates into simple ones, based on the types of substrate available [134]. Primarily, pretreatment methods are classified into four major groups: physical (mechanical pretreatment, extrusion, and pyrolysis), physicochemical (steam explosion, ammonia fiber explosion, CO_2_ explosion, liquid hot water, wet oxidation, sonification, and microwave-based pretreatment), chemical (ozonolysis, acid hydrolysis, alkaline hydrolysis, oxidative delignification, organosolvation, and ionic liquids), and biological (enzymatic hydrolysis) pretreatments. Among these pretreatments, the physicochemical and chemical treatments are the most efficient [135].

The feasibility of H_2_ production using a mixed microbial population is likely to be restricted because of H_2_ consumption by methanogens. Therefore, pretreatment of the biocatalyst parent culture may be beneficial for shifting the metabolic pathways to increase acidogenesis, and to inhibiting methanogenesis to improve the H_2_ production yield with prevention of competitive growth and coexistence of other H_2_ consuming microorganisms [60,79]. The different biocatalyst pretreatment methods include heat shock (temperature, >80 °C), chemical methods (to inhibit specific metabolic functions; 2-bromoethanesulfonic acid, acetylene, Na_2_SO_4_, fluvastatin, chloroform, and iodopropane), acid shock (pH < 4), alkaline shock (pH > 9), an oxygen shock method (oxygen/air), load shock (higher substrate concentration), infrared irradiation, and freezing and thawing (−25 °C for 24 h, followed by a 5 h thaw at 30 °C). The different functional properties of ozone (ozone bubbles) and microwave irradiation methods were evaluated. Indeed, the pretreatments applied to the parent inoculum facilitated the selective enrichment of acidogenic bacteria capable of producing H_2_ as the end product with the simultaneous prevention of hydrogenotrophic methanogens [136]. Because of the physiological differences between the H_2_ producing acidogenic bacteria and the H_2_ consuming bacteria (methanogens), the pretreatment of biocatalysts can also provide a fundamental basis for the development of a H_2_ production system [60,137].

## 6. Conclusions

The multidisciplinary fermentation processes used for the production of H_2_ were numerous, and a variety of substrates were examined. The individual processes possess their own inherent limitations, such as low substrate conversion efficiency, accumulation of VFAs as carbon-rich acid intermediates, and change in system redox conditions and buffering capacity. Thus, to overcome the potential limiting factors and to improve the efficiency of the H_2_ production process, an understanding of the mechanisms of H^+^ reduction, functional roles of membrane components, composition of the communities, development of cultures, and design and development of competent bioreactors are the critical areas for both photo- and dark fermentation processes. The initial research on H_2_ was typically confined to the use of pure cultures as a biocatalyst, and the selection of the biocatalyst depends primarily on the type of fermentable substrates. There is strong consensus that using a mixed microbial population as a biocatalyst is a favorable and practical choice to scale up the technology of H_2_ production, primarily with wastewater as the substrate (carbon source). Additionally, mixed cultures are typically preferred because of the operational ease, stability, diverse biochemical functions, and probability of using a wide variety of substrates. The optimization of the process parameters is clearly necessary to scale up the technology. The residual organic fraction as a soluble fermentation product after acidogenesis is one of the key limiting factors that requires considerable attention. The approaches with integration that use the acid-rich reactor effluents with the simultaneous recovery of energy must be efficient and completely established for the commercialization of the process to be economically feasible.

In conclusion, the basic and applied research on H_2_ production provides additional insight into the process for a better understanding to establish an optimized environment. Although various novel approaches are anticipated in future years to overcome some of the persistent problems, biological H_2_ production technology requires a multidisciplinary approach for the process to be eco-friendly and economically feasible.
